# Pharmacists as Personalized Medicine Experts (PRIME): Experiences Implementing Pharmacist-Led Pharmacogenomic Testing in Primary Care Practices

**DOI:** 10.3390/pharmacy9040201

**Published:** 2021-12-16

**Authors:** Miles J. Luke, Nina Krupetsky, Helen Liu, Clara Korenvain, Natalie Crown, Sameera Toenjes, Beth A. Sproule, Micheline Piquette-Miller, Lisa M. Guirguis, Lisa M. McCarthy

**Affiliations:** 1 Pharmacy Services, Women’s College Hospital, Toronto, ON M5S 1B2, Canada; m.luke@mail.utoronto.ca (M.J.L.); nina.krupetsky@uhn.ca (N.K.); clara.korenvain@wchospital.ca (C.K.); natalie.crown@utoronto.ca (N.C.); sameera.toenjes@mail.utoronto.ca (S.T.); 2 Leslie Dan Faculty of Pharmacy, University of Toronto, Toronto, ON M5S 3M2, Canada; helenchen.liu@mail.utoronto.ca (H.L.); beth.sproule@camh.ca (B.A.S.); m.piquette.miller@utoronto.ca (M.P.-M.); 3 Center for Addiction and Mental Health, Toronto, ON M6J 1H4, Canada; 4 Department of Psychiatry, University of Toronto, Toronto, ON M5T 1R8, Canada; 5 Faculty of Pharmacy and Pharmaceutical Sciences, University of Alberta, Edmonton, AB T6G 1C9, Canada; lguirgui@ualberta.ca; 6 Institute for Better Health, Trillium Health Partners, Mississauga, ON L5B 1B8, Canada; 7 Department of Family and Community Medicine, University of Toronto, Toronto, ON M5G 1V7, Canada; 8 Women’s College Research Institute, Women’s College Hospital, Toronto, ON M5S 1B2, Canada

**Keywords:** pharmacy, pharmacists, pharmacogenomics, service implementation, theoretical domains framework

## Abstract

Research exploring the integration of pharmacogenomics (PGx) testing by pharmacists into their primary care practices (including community pharmacies) has focused on the “external” factors that impact practice implementation. In this study, additional “internal” factors, related to the capabilities, opportunities, and motivations of pharmacists that influence their ability to implement PGx testing, were analyzed. Semi-structured interview data from the Pharmacists as Personalized Medicine Experts (PRIME) study, which examined the barriers and facilitators to implementing PGx testing by pharmacists into primary care practice, were analyzed. Through thematic analysis, using the theoretical domains framework (TDF) domains as deductive codes, the authors identified the most relevant TDF domains and applied the behavioural change wheel (BCW) to generate intervention types to aid in the implementation of PGx testing. Pharmacists described how their professional identities, practice environments, self-confidence, and beliefs in the benefits of PGx impacted their ability to provide a PGx-testing service. Potential interventions to improve the implementation of the PGx service included preparing pharmacists for managing an increased patient load, helping pharmacists navigate the software and technology requirements associated with the PGx service, and streamlining workflows and documentation requirements. As interest in the wide-scale implementation of PGx testing through community pharmacies grows, additional strategies need to address the “internal” factors that influence the ability of pharmacists to integrate testing into their practices.

## 1. Introduction

Pharmacogenomics (PGx) relates to the interplay between an individual’s genome and their response to medications [1,2]. The first step in applying PGx principles is genetic testing; once done, the premise is that healthcare providers can then better predict how an individual will respond to certain medications. As medication experts, pharmacists are well-positioned to implement pharmacogenetic testing, make PGx-based prescribing recommendations, and educate patients and other healthcare professionals about how to interpret the results of pharmacogenetic tests [3]. Despite this, barriers to the implementation of PGx in primary care practice include the cost of the service, lack of time, gaps in the knowledge of pharmacists, and a lack of patient motivation [4,5,6]. The Pharmacists as Personalized Medicine Experts (PRIME) study aimed to address many of these gaps by providing participating pharmacists with a PGx training program, and it then supported these pharmacists as they began offering PGx services in their existing practices [7].

Providing PGx services requires that pharmacists perform new tasks within the complex contexts of their existing practice settings. Implementation research can be used to understand the interplay between these new tasks, or the behaviours and the factors that influence the participation of pharmacists in them. Prominent conceptual frameworks used in conducting implementation research include the theoretical domains framework (version 2) (TDF), and the consolidated framework for implementation research (CFIR) [8,9]. The TDF can guide the identification of the determinants of service implementation that exist at multiple levels (e.g., the individual level, the organizational level, and the health-system level), however, the majority of its 14 domains relate to factors that govern behaviour often conventionally considered to occur at an individual level (i.e., those that are “internal” to the actors implementing and delivering the new service or program) [8,10]. A unique feature of the TDF is that it complements the capability, opportunity, motivation, behaviour (COM-B) model for understanding behaviour [11]. The COM-B model suggests that any given behaviour results from the intersection of a person’s physical and psychological abilities to perform the behaviour (capability), the social and environmental factors that make the behaviour possible (opportunity), and their beliefs and emotions that stimulate the behaviour (motivation) (for additional details, see Cane et al., 2012) [8]. The domains of the TDF have been mapped onto the components of the COM-B model, each of which represents antecedents for behaviour and is, thus, a potential target for interventions aimed at promoting behaviour change [8,11]. The behaviour change wheel (BCW) incorporates the COM-B model and offers a theory-based tool for determining effective intervention strategies and policies for changing a given behaviour [11].

Like the TDF, the CFIR is a useful tool for understanding the determinants of implementation; however, the concepts included in the CFIR primarily (but not exclusively) focus on the determinants that act at an organizational or health-system level [9,10]. One could argue that CFIR focuses on barriers and facilitators that are “external” to the pharmacists who are implementing the services, such as the existence of appropriate reimbursement schedules, lack of time, and the completion of responsibilities [4,5,6,12,13,14].

PRIME pharmacists experienced varying levels of success in implementing PGx services in their community pharmacies and primary care clinic-based practices. In our evaluation of the PRIME training program, which used CFIR as a sensitizing framework for the interview analysis, a key finding was that pharmacists described a lack of self-efficacy as a potential barrier when recruiting patients to the study [7]. In this study, we used the TDF as a conceptual model for further exploring the “internal” experiences of PRIME pharmacists that facilitate and hinder the successful implementation of a novel PGx service. Our aims were to further elucidate the factors influencing the integration of PGx testing by pharmacists in their practices and to use the BCW approach to inform future intervention options to support pharmacists with this integration.

## 2. Materials and Methods

This study was a secondary analysis using a qualitative descriptive approach to data from semi-structured interviews with pharmacists who participated in the PRIME study. The findings of this study are reported according to the Standards for Reporting Qualitative Research (SRQR) [15]. This study was approved by the Women’s College Hospital Research Ethics Board (Protocol 2016-0128E, approved 15 December 2016).

### 2.1. Population

Pharmacists were eligible for participation in interviews if they participated in PRIME. The eligibility criteria from the PRIME study also applied: pharmacists had to have been registered under Part A with the Ontario College of Pharmacists (i.e., permitted to provide patient care) and practicing at least 50% of the total work hours per month in primary care settings, including community pharmacies and interprofessional primary care clinics (referred to as “family health teams” or “nurse-practitioner-led clinics” in our jurisdiction) [7]. Pharmacists were excluded if they worked primarily in hospital pharmacies or if they had undertaken another pharmacogenomics training program. Participants were also required to speak English, as this was the language of the interviewer.

### 2.2. Recruitment Strategy

All study pharmacists who completed the PRIME training program were invited to participate in telephone interviews through an emailed invitation and a study information letter. Potential participants who expressed interest were contacted by telephone to answer any questions regarding the study information letter and to obtain verbal consent before initiating the interview.

### 2.3. Data Collection

Semi-structured telephone interviews were conducted between February and April 2017. Data collection continued until all interested PRIME pharmacists had been interviewed. The interview guide aimed to obtain an in-depth description of the pharmacists’ perceptions and experiences with providing PGx testing in their practice, particularly focusing on the barriers and facilitators. The interview questions were iteratively refined as the interviews progressed. The interviews were audio recorded and transcribed verbatim by an independent transcriptionist. The transcriptions were then verified by the interviewer and de-identified.

### 2.4. Analysis

Braun and Clark’s approach to reflexive thematic analysis was used to code, interpret, and analyze the de-identified interview data [16]. The 14 domains of the theoretical domains framework (TDF) were applied as deductive codes to the interview data to describe the subjective accounts of the participants’ experiences with PGx testing in practice [17].

An initial coding manual was prepared by M.L. to specify how the TDF would be applied to the interview data and to provide consistency in our analysis. The manual was independently verified by L.M. for congruency with the TDF and to ensure relevance to the research question. M.L. and H.L. then independently applied the coding manual to the first three transcripts, meeting after each transcript to discuss and make revisions to the manual as necessary. The remaining seven transcripts were coded by M.L. and H.L. without further discussion.

All coded data was reviewed by M.L., H.L., and L.M., and any discrepancies were discussed and reconciled. The analysis then focused on identifying the most relevant theoretical domains (i.e., factors that most strongly influence behaviour) by determining those with multiple or conflicting perspectives present, with evidence of strong ideas or beliefs that could significantly influence the delivery of PGx services, and/or a high frequency across transcripts [18]. These criteria were applied to coded domains by M.L. and H.L. independently, and then a consensus list of the key theoretical domains was obtained through discussion. Finally, a similar process was used by M.L. and H.L. to independently identify, and then discuss, semantic themes, defined as the explicit patterns of similar concepts in what the interview participants said, within the key theoretical domains. Themes relating to the barriers and facilitators to implementing PGx services in pharmacy primary care practice were then mapped onto the corresponding components of the COM-B framework [11].

### 2.5. Reflexivity

Reflexivity was used to acknowledge the researchers’ underlying biases and to facilitate an understanding of how they influenced the study design and the interpretation of the data [19]. Before beginning analysis of the interview data, the investigators primarily involved in the data analysis (M.L. and H.L.) prepared statements of reflexivity that described their views and beliefs around pharmacy practice in primary care. Briefly, both researchers shared a desire for expanded scopes of services for pharmacists practicing in the community, and for a greater involvement in the clinical aspects (rather than drug distribution) of patient care. Given both their experiences practicing in busy community pharmacies and M.L.’s experience as a pharmacy resident on an interprofessional primary care team, both researchers expected primary care clinic pharmacists to have a much greater capacity to introduce new time-intensive services into their practices than community pharmacists. However, both researchers also shared the belief that community pharmacists are highly trained medication experts who could support a novel PGx service with additional support and/or changes to their workflows. Given our positionality regarding the barriers faced in busy community pharmacies, our thematic analysis may have been more sensitive to interview data confirming or refuting these barriers. However, we believe that our use of the TDF to guide our analysis and a third investigator to review the coding ensured the quality of the analysis.

## 3. Results

### 3.1. Participants

A total of 10 of the 21 pharmacists who completed the PRIME training program responded to the email invitation and partook in interviews. Five of these participants practiced in community pharmacy, four worked in interprofessional primary care clinics, and one participant worked in both of these settings. These practice sites were located across Ontario, Canada, including both urban and rural settings.

### 3.2. Summary of Findings

The PRIME pharmacists discussed concepts relating to all 14 of the TDF domains during the interviews. The key theoretical domains that were identified on the basis of the content and patterns observed in the dataset were: (1) Memory, Attention, and Decision Processes; (2) Behavioural Regulation; (3) Social Influences; (4) Environmental Context and Resources; (5) Social/Professional Role and Identity; (6) Beliefs about Capabilities; and (7) Beliefs about Consequences. Themes within these key TDF domains were identified and are presented in the following sections as they relate to the components of the COM-B model (capability, opportunity, and motivation) (Table 1).

### 3.3. Capability

TDF domains relating to the psychological “capability” component of the COM-B model that were prominent in our dataset included Memory, Attention, and Decision Processes, and Behavioural Regulation.

Pertaining to Memory, Attention, and Decision Processes, pharmacists described the delivery of PGx services as a complex process involving numerous discrete steps and documentation requirements, all of which felt cumbersome for the pharmacists and patients. Overall, some pharmacists found it difficult to manage higher patient loads, and some patients lost interest in completing PGx testing at various points in the process. Not all pharmacists were explicit about which components of the service added complexity; as such, it is unclear if delivering the PGx service in the context of the PRIME study (with additional documentation and reporting requirements) was what caused the complexity, or if it was the PGx service itself. There was similar ambiguity regarding whether patients lost interest in completing the symptom questionnaires and other documentation for the study, or if there was waning motivation over time to follow up with pharmacists about the clinical implications of their PGx test results.

Relating to Behavioural Regulation, the pharmacists described mechanisms they developed or adapted in order to facilitate the completion of the behaviours involved in the delivery of the PGx service. These included using the existing electronic medical record (EMR) software of their practices to schedule patient appointments, downloading mobile applications to aid in patient and data tracking, and sending symptom questionnaires to patients ahead of appointments to facilitate monitoring. Several pharmacists also promoted the PGx services to prescribers and other healthcare workers within their networks to generate patient referrals into the service.

### 3.4. Opportunity

Social Influences, and Environmental Context and Resources were also determined to be key TDF domains in our dataset.

Two themes were identified within Social Influences. Pharmacists described: (1) Building and leveraging relationships with patients to facilitate patient recruitment in the PGx service; and (2) The impact of relationships within their personal network of healthcare providers on the success of PGx service implementation.

In terms of recruiting patients, pharmacists found patients were generally interested in undergoing genetic testing for the purposes of optimizing their pharmacotherapy. However, pharmacists considered patients’ concerns regarding consent, privacy, and the ownership of their genetic information as barriers to patient recruitment. Pharmacists experienced challenges with the recruitment and follow-up of people who were less well known to the pharmacists and attributed the low engagement to low patient motivation as part of mental health conditions and discomfort in interacting with unfamiliar healthcare providers. Conversely, pharmacists reported success in recruiting patients with whom they had pre-existing relationships. Pharmacists described the cultivating of trust between the patient and pharmacist as helpful to overcoming some patient-specific barriers. Patient–pharmacist relationships led to greater patient willingness to enroll and participate in the PGx service.

Similarly, pharmacists found that the success of the PGx service was influenced by the network of healthcare providers with whom they worked. Some pharmacists worked closely with physicians who were unaware of the possible applications of PGx testing in guiding prescribing decisions, resulting in few or no referrals for PGx testing from them. Some pharmacists increased awareness of the PGx service by hosting “lunch and learn” presentations, or by recruiting patients themselves and demonstrating the potential benefits of PGx to those patients’ prescribers. As awareness increased, so too did referrals from prescribers. Pharmacists working in interprofessional primary care clinics contrasted their success working with the clinic’s family doctors against their difficulties working with external psychiatrists, who were not as accessible or willing to act on pharmacists’ PGx-based recommendations because of the lack of an existing working relationship. One pharmacist described having customized their approaches to effective verbal and written communication with the physicians in their area to align with physician preferences, learned through previous encounters with those physicians (e.g., pharmacotherapy recommendations were more acceptable to physicians when presented with straightforward actions, rather than when they included descriptions of detailed evidence). This strategy facilitated the physician buy-in to the PGx service and the acceptance of their PGx-based recommendations.

Within the domain, Environmental Context and Resources, pharmacists commented on the fit of the PGx service into their existing workflow. Pharmacists from both practice settings reported insufficient time, competing work priorities, and inadequate staffing as barriers to implementing PGx services. Barriers to PGx services also differed with respect to practice settings. Pharmacists in the community reported dispensing prescriptions as a barrier, and pharmacists in primary care clinics cited their involvement in other programs offered at their practice setting (e.g., smoking cessation, warfarin monitoring). Common across both practice settings was pharmacists reporting working longer hours to accommodate the integration of the PGx service into their practice.

Conversely, some pharmacists described features of their existing workflows that facilitated the incorporation of PGx services into their practices. One community pharmacist described their practice as “the new model of pharmacy where you actually spend the time with the patient, getting to know them, being one of their primary healthcare providers” (Participant 981). As such, the assessments, counseling, and care planning involved in the PGx service aligned closely with the existing services offered by the pharmacist. Similarly, some primary care clinic pharmacists noted that PGx-related behaviours (e.g., identifying drug therapy problems, or providing pharmacotherapy recommendations to prescribers) were consistent with the care they routinely provided. For these pharmacists, pharmacogenetics was not an added responsibility but, rather, a tool they used to better inform their clinical activities. Furthermore, the appointment-based scheduling of the primary care clinic-based pharmacists’ patient interactions ensured that their workload remained manageable.

### 3.5. Motivation

Three key theoretical domains mapped onto the “motivation” component of the COM-B model: Social/Professional Role and Identity; Beliefs about Consequences; and Beliefs about Capabilities.

A common motivator for the pharmacists’ delivery of PGx services was their belief that the use of PGx will become more widespread in the future, and the leadership role they expect pharmacists to play in its growth (i.e., Social/Professional Role and Identity). They shared a desire for pharmacists to support other healthcare providers implementing PGx services and making PGx-based prescribing decisions. Pharmacists recognized the potential for the significant improvements in patient outcomes that PGx could provide (Beliefs about Consequences). They viewed PGx as an opportunity for them to add value by tailoring pharmacotherapy to the genetic phenotypes of individual patients. Some pharmacists practicing in the community were also motivated to implement PGx services as a strategic business decision in order to increase patronage at their pharmacy.

On the other hand, some pharmacists spoke candidly about a lack of confidence in their knowledge and abilities as a barrier to recruiting patients for the PGx service (Beliefs about Capabilities). Some pharmacists remarked that they felt confident upon completing the PRIME training program, but that this confidence waned as time passed and that the knowledge and skills acquired in the program became more difficult to recall. Regardless of their levels of confidence in their capabilities, multiple pharmacists reported a desire for additional training in pharmacogenomics, or a “refresher course”, to make them feel more comfortable recruiting patients into the program.

## 4. Discussion

In this study, which involved a secondary analysis of interviews with pharmacists, the application of the TDF framework revealed “internal” facilitators and barriers with respect to integrating PGx testing in pharmacy practice. Successful implementation was dependent on features of the pharmacists’ professional identities, practice environments, self-confidence, and the perceived benefits of adding PGx testing to their practice.

Previous work identified a lack of pharmacogenomic education and knowledge as key barriers preventing the uptake of PGx services into primary care pharmacy practices [12,20,21,22,23,24]. The PRIME training program addressed these barriers by providing pharmacist participants with both theoretical and practical knowledge for applying PGx in practice, such as drug–enzyme pairings and how to effectively communicate about PGx with patients, respectively. However, possibly because PRIME did not specifically address how participants would integrate the study procedures into their existing practice settings and workflows, pharmacists reported logistical and administrative gaps in their capabilities to deliver PGx services. Thus, future interventions may be more successful if they were designed to prepare pharmacists for managing an increased patient load, for navigating any software and technology requirements associated with the PGx service, and for streamlining workflows and documentation requirements as much as possible (e.g., sending patients symptom questionnaires to be completed in advance, or conducting follow-up via telephone rather than in person).

Incorporating this practical learning into the curriculum of a PGx training program aligns with the “education”, “training”, and “modelling” intervention types suggested by Michie, Atkins, and West (2014) to address the behavioural barriers relating to the TDF domains, Memory, Attention, and Decision Processes, and Behavioural Regulation [11]. An alternative intervention type would be to address these logistical gaps through “enablement”, wherein, for example, the training program faculty would assume an active role in the administrative aspects of the service (e.g., scheduling appointments, administering symptom questionnaires, creating templates for clinical documentation, etc.). Doing so would enable pharmacists to focus solely on the clinical aspects of the PGx service.

Similarly, “enablement” and “environmental restructuring” would be appropriate interventions for addressing the pharmacists’ concerns around environmental contexts and resources [11]. Specific strategies are inherently unique to the practice setting: pharmacists in interprofessional primary care clinics may require adjustments to their schedule so that they are allotted time to specifically devote to the PGx service; community pharmacists may benefit from larger-scale changes, such as additional staffing and automated on-screen prompts for staff to inquire about the interest of eligible patients in PGx testing.

Interestingly, some community pharmacists who reported minimal difficulty in incorporating the PGx service into their existing workflows described practice settings with additional support for medication dispensing. Likewise, the Innovative Canadian Pharmacogenomic Screening Initiative in Community Pharmacy (ICANPIC) study (2017), which demonstrated the feasibility of a comprehensive PGx service in two community pharmacies that offered a suite of “professional” (i.e., nondispensing) services and were “adequately staffed to balance dispensing responsibilities with clinical pharmacy activities” [25].

Another option for environmental restructuring would be creating a reimbursement schedule that provides a funding mechanism for pharmacies for providing PGx-based testing and consultations [13]. Breaux et al. (2021) suggest that the community-pharmacy-based delivery of PGx services is cost-effective and holds the potential for cost savings [26]. Green Shield Canada (GSC) is a health benefits provider that has acted on these preliminary findings by creating a new program to reimburse pharmacists for facilitating PGx testing and for providing counselling to GSC health plan members not responding to, or experiencing adverse effects from, their current drug therapy for generalized anxiety disorder and major depressive disorder [27]. GSC intends to eventually expand this program to other conditions; to date, there has been limited economic analysis of PGx, and further research may be needed to support the widespread adoption of such reimbursement schedules.

The motivation of pharmacists to apply PGx services was bolstered by their belief in PGx as a valuable addition to the professional role of pharmacists, and as beneficial to patient health. Pharmacists who participated in this research may have inherent positive beliefs and motivations. Future research measuring the presence of these beliefs would determine the value in adding “persuasion” or “modelling” interventions to similar PGx training programs for pharmacists [11]. Modelling (e.g., stories of successful implementation in community practice) may also address barriers in the Social Influences and Behavioural Regulation domains.

### 4.1. Limitations

Of the 21 pharmacists who participated in the PRIME PGx training program, only 10 responded to emails asking them about their interest in participating in interviews about their experiences implementing PGx in practice. This allows for the possibility that not all relevant data were uncovered during the interviews. However, equal representation of pharmacists working in different primary care practice settings (i.e., community pharmacies and interprofessional-team-based clinics) was achieved, allowing for the consideration of a diverse range of barriers and facilitators affecting the implementation of PGx services in variable contexts.

One might contend that studies employing the TDF to explore a phenomenon would ideally use the theoretical framework not only for analysis but also to guide the study design and data collection [17]. In this paper, we describe a secondary analysis using the TDF that was not planned at the time that the interviews with the PRIME pharmacists were conducted. Therefore, the interviews were not explicitly designed to solicit responses directly relating to TDF domains, possibly resulting in the omission of data that would have further informed the analysis (e.g., pharmacists described the delivery of the PGx service as a complex process, but interviews did not probe into which components of the service added complexity). That said, the interviews included many open-ended prompts that invited the participants to discuss all aspects of the program implementation that they felt were most important. These factors, as well as a preliminary appraisal of the interview transcripts to assess the fit of our data to the TDF, were considered when designing the proposed study and were acknowledged during our analysis.

### 4.2. Future Research

Additional opportunities for future research include evaluating the viability of intervention types and the specific interventions described by the COM-B model and the pharmacist participants, respectively. Criteria, such as APEASE (affordability, practicability, effectiveness and cost-effectiveness, acceptability, side-effects and safety, equity), can be used to evaluate the promise of future interventions that can then be implemented and evaluated [11]. Such interventions could be incorporated into existing PGx training programs, such as those created by Breaux et al. or the PRIME group, and then studied iteratively for their effects on the successful implementation of pharmacist-led PGx services [7,26].

## 5. Conclusions

Pharmacists described how their professional identities, professional relationships, practice environments, self-confidence, and belief in the benefits of PGx influenced the degree of success they experienced in performing the complex processes involved in the PGx service. Given the inconsistent uptake of the PGx service across the participating pharmacists, our findings suggest that pharmacists must possess a combination of these factors for implementation to be successful. The TDF model was used to articulate the barriers and facilitators of service implementation encountered by these pharmacists. Enablement, environmental restructuring, and persuasion are the potential intervention types suggested by the BCW to address these barriers and equip primary care pharmacists with the capability, opportunity, and motivation to implement and deliver a PGx service in their practice.

## Figures and Tables

**Table 1:** Quotations of pharmacists that are representative of themes identified in the interview data.

TDF Domain	Theme	Representative Quotations
CAPABILITY		
Memory, Attention, and Decision Processes	Providing PGx-based interventions is a complex process	“So in that moment when you’re with the patient and you’re trying to think on your toes about all the other things you have to do as a pharmacist, you also have to think, okay, what form do I have to get them to fill in now? And then if I hadn’t done it in a couple of weeks because I was so busy, I’d kind of forget […].” [Participant 599]
Behavioural Regulation	Mechanisms to facilitate behaviours involved in the delivery of the PGx service	“I started doing things that make it easier for myself though as far as flow goes. […] So at least recording when I’ve approached the patient. Because that was part of it, right, is the initial flow sheet when you’ve approached someone, whether or not they’ve agreed or not to continue with the process. […] I also make a list for myself obviously of all the PRIME patients and when I’ve actually got the consent done. So it was just kind of figuring out how I was going to do all of that at the beginning. So it’s definitely a lot easier now.” [Participant 288] “The people who have joined the study through me have been referred to me by the nurse practitioners. I did a lunch and learn with them when we did the training and it was implemented to give them a low down on the type of patients that we were looking for, and all of that. So, they’ve been sending them to me through the EMR message system to contact and then get the permission done, and all that good stuff.” [Participant 701]
OPPORTUNITY		
Social Influences	Relationships with patients influence their enrollment in the PGx service	“In the family health team setting, I didn’t have a direct relationship with the individuals. I hadn’t seen them for any other reason. In the community pharmacy, it was different. I knew these individuals very well over the years. Did that change how I recruited them? It may be a little bit more convincing that, you know, it just might be something they might find the value of. In the community pharmacy, that relationship kind of helped make it an easy transition.” [Participant 432]
	Personal network of healthcare providers influences the success of PGx service implementation	“[…] it’s harder for us to enroll patients if there aren’t more referrals from the physicians and they don’t see a benefit in it. So, if there is a way of getting physicians to see a benefit in the program, I think it will be easier to get more patients enrolled in this type of a program.” [Participant 517] “I found it’s much easier for me to work with the physicians in the family health team who are accessible to me, that I can send a message through the EMR [Electronic Medical Record] or I can catch them in person and talk to them, than it is to be able to work with one of the psychiatrists. […] So, I mean as soon as I saw a patient and we had the [test] results for some of them, I was able to make changes within a day or within that day just because I had accessibility to the physician. And with psychiatry, I mean my one patient, she doesn’t have her appointment until the middle of March, you know. I’m not sure when they’ll actually make changes. “ [Pharmacist 288]
Environmental Context and Resources	Fit of PGx services into pharmacists’ existing workflows	“And again, it’s not like the interpretation of the clinical stuff, it’s just the process just needs to be catered to the nature of the way pharmacy is right now. Which is: we’re still tied to checking prescriptions, right? So, I mean if I had a technician in then maybe the pharmacist would be freed up. […] But just for my situation, I would love to incorporate it in the future. It just would have to fit right into the operations of the pharmacy.” [Participant 599] “So, what I thought was interesting in this study is it really is looking at how feasible is it for pharmacists to perform these new kind of… I mean it’s still within our counselling and we’re doing it anyways. But it’s a different field.” [Participant 396] “So, getting it into my workflow in the style of pharmacy I have is quite easy because I am busy for periods of time but I will take that half hour if I need to with a patient, and make up my fill times for prescriptions afterwards. So for me, it was an easy fit into my workflow because I was having open and honest conversations with a lot of my patients.” [Participant 981]
MOTIVATION		
Social/Professional Role and Identity	Pharmacogenomics as a field of growing importance for pharmacists to be involved in	“I think that it’s potentially something that will be very prominent in the future as to how we select a medication to personalize therapy. And I wanted to be on the ground level to have a good experience from the very beginning.” [Participant 598] “I think for me it was more also just a bit of it was explaining the pharmacy role—right, what else can pharmacists demonstrate that they can do as well. So that was probably more of a motivation than the pharmacogenetics itself.” [Participant 396] “As an owner I just saw it as a great opportunity to offer a value-added service so that people know that we’re forward thinking and really customizing their care.” [Participant 888]
Beliefs about Capabilities	Lack of confidence in knowledge/abilities as a barrier to recruiting patients	“I feel that I definitely have a better understanding than I had before. I definitely have more resources that I’ve learned about through this process. So if I were to be asked a question, I think I can be able to find the right resources to provide some opinion. […] But I wouldn’t necessarily consider myself an expert or anything like that… if I felt more competent with it, then maybe I would prioritize that more in terms of my time to look for these patients.” [Participant 396]
Beliefs about Consequences	PGx offers potential improvements in patient outcomes	“Yeah, there’s a few patients that were […] really hesitating [to start a medication] because of either previous experiences with side effects with medications or are really hesitant because they’ve only heard or read the drug profile, which is often quite negative when it comes to adverse events and things like that. So having that study, explaining how things may work in your body, which ones may not affect you or cause as many side effects, I think just provided a little bit more assurance to the patient. So, I’ve had two of those patients who actually were willing to resume therapy because of the study. I think it makes them feel more empowered.” [Participant 396] “When I first signed up, I would say it was probably just another service that I thought I could offer to them. I wanted to see what kind of place it would have in the pharmacy. But after doing the study, like the training and now incorporating it, I see a value in it and it does interest me. I feel like it could give me more information and an edge in terms of being able to treat patients.” [Participant 888]

## Data Availability

The data presented in this study are available upon request from the corresponding author. The data are not publicly available in order to maintain the privacy of the study participants.
